# The Physical Demands of Match-Play in Academy and Senior Soccer Players from the Scottish Premiership

**DOI:** 10.3390/sports10100150

**Published:** 2022-10-08

**Authors:** Ryland Morgans, Eduard Bezuglov, Patrick Orme, Kyler Burns, Dave Rhodes, John Babraj, Rocco Di Michele, Rafael Franco Soares Oliveira

**Affiliations:** 1 Department of Sports Medicine and Medical Rehabilitation, Sechenov State Medical University Moscow, 119991 Moscow, Russia; 2 High Performance Sports Laboratory, Moscow Witte University, 115432 Moscow, Russia; 3 Sport Science and Medical Department, Bristol City FC, Bristol BS3 2EJ, UK; 4 Sport Science and Medical Department, Dundee United FC, Dundee DD3 7JW, UK; 5 Football Performance Hub, School of Sport and Health Sciences, University of Central Lancashire, Preston PR1 2HE, UK; 6 Division of Sport and Exercise Sciences, School of Applied Sciences, Abertay University, Dundee DD1 1HG, UK; 7 Department of Biomedical and Neuromotor Sciences, University of Bologna, 40126 Bologna, Italy; 8 Sports Science School of Rio Maior-Polytechnic Institute of Santarém, 2040-413 Rio Maior, Portugal; 9 Life Quality Research Centre, 2040-413 Rio Maior, Portugal; 10 Research Centre in Sport Sciences, Health Sciences and Human Development, 5001-801 Vila Real, Portugal

**Keywords:** physical performance, match-play, elite European soccer players, competitive level

## Abstract

The present study aimed to assess the physical match performance among senior and youth soccer players from an elite Scottish Premiership club during the 2021/2022 season. Twenty-two first team (25.9 ± 4.5 years, 78.3 ± 8.2 kg, 1.82 ± 0.07 cm) and 16 youth players (16.8 ± 0.9 years, 70.1 ± 6.8 kg, 177 ± 5.8 cm) were examined. A selection of physical match performance variables were measured using a global positioning system. Linear mixed-effect regressions revealed for all examined variables no significant differences between first team and U-18 players and no significant differences between playing level by position interaction. Across both teams, Centre Backs compared to Wing Backs, showed a 295 m (*p* < 0.01) lower high-intensity distance, and performed on average 36 fewer very-high intensity decelerations (*p* = 0.03). Comparing to Wide Midfielders, Centre Backs showed lower total (1297 m, *p* = 0.01), high-intensity (350 m, *p* = 0.01), and sprint (167 m, *p* < 0.01) distances. Sprint distance was also lower in Centre Backs vs. Strikers (118 m, *p* = 0.03), and in Central Midfielders vs. both Strikers (104 m, *p* = 0.03) and Wide Midfielders (154 m, *p* = 0.01). The present findings highlight the physical match performance of elite Scottish players and provide useful information within the context of understanding how methods of physical development of youth soccer are implemented in different countries.

## 1. Introduction

Soccer is a physically demanding sport that is played throughout the world by a broad range of individuals [1]. The occurrence of powerful activities during match-play has increased significantly over the last few decades [2]. The evolution of these studies has aligned with advancements in technology and methods that have enhanced the ability of practitioners to quantify these aspects of physical performance during soccer training and match-play [3]. However, the existing literature has primarily studied whole match data with limited evidence analyzing mean (e.g., without considering match outcome) and peak periods (e.g., usually defined as the most intense periods of a match) that often exposes players to the highest external loads within the match [4,5].

The most intense periods during match-play have recently been referred to as ‘worst case scenarios’ [6,7,8]. Providing a greater understanding of the maximal physical demands during specific time periods during match-play may allow coaches and practitioners to prepare players more appropriately for these high intensities [9]. While different methods exist to quantify these periods [6], the overall concept is similar where there is a need to identify the most intense periods of match-play to re-create the same physical stimulus in training in order to reach a positive physiological outcome [10]. While previous research has reported that players do not achieve peak physical demands during match-play [4], from a practical perspective, a greater understanding of these key physical match periods (mean and peak) and the potential differences between youth (U-18) and senior (first team) teams from within the same club may allow coaching staff to design and deliver more integrated, age-specific sessions. A simple way to identify peak demands could be to quantify the most intense variables of the match, such as high-intensity distance (also known as high-speed running distance), sprint distance, accelerations, and decelerations, as previously suggested in a systematic review of load measures in soccer [11]. Usually, these variables are collected by a Global Positioning System (GPS) or other high-technology equipment [12] that consistently track activity variables produced by a player during training sessions or match-play [13].

At the elite level, soccer is played at various age groups ranging from developing academy youth players through to senior first team players (~>18 years old). The demands of soccer may vary significantly in relation to a number of factors, such as the players’ age, the players’ competitive level, and the playing style of any given team [14,15]. In some cases, the playing style may be linked to the playing culture within certain countries. In turn, this may affect the physical demands of soccer match-play [16].

An important part of developing soccer players for elite-level competition is ensuring they are able to cope with the physical demands of senior elite-level soccer. With this in mind, it is important to fully understand the differences in required physical outputs between late-stage academy soccer (U-18) and senior soccer, as suggested by a recent systematic review on training intensity in young male soccer players [17]. This may provide practitioners with a better understanding of the appropriate methods of physical development for elite academy players. In terms of developing soccer players, it may, therefore, be important to understand the differences in the physical performance during training and match-play in both elite youth and elite senior players to allow practitioners to appropriately inform this process. Previously, Buchheit et al. [18] presented data to demonstrate the physical match performance of elite soccer players from U-13 to U-18 age groups. This study provides important insight into the match demands placed on these players but does not provide an understanding of the demands undertaken during training sessions throughout the season. Morgan et al. [19] also recently found that players undertake an average of 305 change of direction (COD) actions during an elite match in English youth soccer. Kai et al. [20] also reported that the number of COD actions performed on average by developing players in the Japanese University League was only 183. Furthermore, Di Salvo et al. [21] provided data examining high-intensity actions during senior soccer match-play, however, Bradley et al. [22] presented more recent data from various competitive levels, which concluded there was significant variation in the physical demands as a result of playing standards. This specific research area warrants further investigation.

To date, no study has examined and compared the mean and peak physical performance characteristics in match-play of youth and senior soccer players from the same Scottish Premiership (SP) club. Historically, the SP league is known for physically challenging match-play and there has been an increasing competitive level over recent years. Examining data from different countries and leagues is vital to improve our understanding of various methods of physical development [23]. Albeit research regarding physical demands in youth soccer has grown recently, more is warranted to fully understand the complexities of developing players and comparisons with their senior counterparts.

Several physical variables have previously been reported to be critical moments in successful performance and often differentiate between high- and low-level adult players [14]. As an example, a recent study compared U-17, U-19, and the first teams from a Danish SuperLiga club and found a higher number of accelerations and decelerations for the U-19 when compared with both the U-17 and the first team, while no differences were found for distance covered during high-intensity running or sprinting [24]. However, it is important to fully understand the mean and peak periods of physical performance during match-play and to identify any differences between competition levels and playing position to provide practitioners with detailed information to allow specific sessions to be designed and delivered. Furthermore, it was earlier evident that physical match demands were different between playing positions in professional players [25], and recently, a systematic review documented that running distances covered in matches are dependent on playing position [26].

Therefore, the aim of this study was to analyze a selection of physical performance indicators in match-play of elite youth and senior soccer players from an SP club. Besides comparing the physical performance between competition levels (youth versus senior), differences between playing positions were also examined. Our hypothesis was that senior, first team players will produce higher physical match performance when compared to their U-18 counterparts. Furthermore, more attacking players will demonstrate greater high-intensity and sprint actions regardless of competitive level.

## 2. Materials and Methods

### 2.1. Participants

Thirty-eight professional senior and U-18 outfield soccer players from a SP club formed the sample for this study. Data from the complete 2021–2022 season highlighted 22 senior outfield players (first team squad) and 16 youth outfield players (U-18 youth squad). Data from the first and U-18 youth teams from the same SP club were analyzed for comparison between senior and youth players, and the influence of playing position between the levels was also examined. The following playing positions were examined: Centre Backs (CB), Wing Backs (WB), Centre Midfielders (CM), Wide Midfielders (WM), and Strikers (ST). Goalkeepers were excluded from the investigation due to the specific nature of their match activity and their low running demands [27,28]. Table 1 shows the age, anthropometric, and playing position characteristics of the examined sample of players. Significant differences between squads were observed for mean body-mass (*p* < 0.01) and mean height (*p* = 0.02), with higher values in first team when compared to U-18 players for both variables. The study was conducted according to the requirements of the Declaration of Helsinki and was approved by the local Ethics Committee of Sechenov University (N 22-21 dated 12 December 2021) and the SP club from which the participants volunteered [29]. To ensure confidentiality, all data were anonymized prior to analysis. Written informed consent was obtained from all participants. Informed written consent was also provided by the parents of participants under 18 years of age.

### 2.2. Procedures

The SP league is typically split into two distinct periods. The first phase can be subdivided into pre-season (average 4-weeks) starting mid-June and competition (25-weeks) concluding in end-December, fulfilling on average 20 official league fixtures. The second phase commences after a 3-week winter break (end-December to mid-January) and also has two definite periods consisting of pre-season (2-weeks) commencing early-January and competition (17-weeks) finishing in mid-May, completing the remaining 18 league fixtures. The Club Academy Scotland (CAS) youth league follows a similar structure to the SP, and is also divided into two phases, before and after a 5-week mid-season break (mid-December to mid-January). The youth pre-season commences mid-June (average 4-weeks) and completes 14 matches until mid-December. The second period re-starts in mid-January and finalizes at end-May completing the remaining 14 league matches. All SP (*n* = 38) and CAS (*n* = 28) youth league matches for the 2021/2022 season were used in the present study, and individual players’ data were included only when that player completed a whole match. For both the first team and U-18 squads, only players having played during the 2021/2022 season at least two whole official matches, respectively in the SP and CAS youth league, were included. The total number of examined individual match performances was *n* = 275 and *n* = 217 for first team and U-18 team, respectively. All data collected resulted from normal analytical procedures regarding player monitoring over the competitive season, nevertheless, approval for the study from the club was obtained [29].

### 2.3. Data Collection

Physical match performance data were monitored during all examined official matches using an 18 Hz GPS technology tracking system (Apex Pod, 50 gr, 88 × 33 mm; Statsports; Northern Ireland, UK), that has been previously validated [30]. All devices were activated 30-min before data collection to allow acquisition of satellite signals and to synchronize the GPS clock with the satellite’s atomic clock [31]. Quantifying the devices’ accuracy indicated a 2.5% estimation error in distance covered, with accuracy improving as the distance covered increased and the speed of movement decreased [32]. To avoid inter-unit error, each participant wore the same device during the study period [33,34], although the present GPS system has been previously reported to have excellent inter-unit reliability [35]. Specifically designed vests were used to hold the devices, located on the player’s upper torso, and anatomically adjusted to each player, as previously described [36]. On completion of each match, GPS data were extracted using proprietary software (Statsports Software; Northern Ireland, UK). Statsports provided written permission to allow all data to be used for research purposes. After data extraction, the exact time points of match kick-off, end of 1st half, start of 2nd half, and match finish were individuated into Statsports software to determine the GPS metrics associated with the playing time.

The total distance covered (m), high-intensity distance (m; total distance covered 5.5–7 m/s), and sprint distance (m; total distance covered >7 m/s) were examined based on previous studies [37,38,39]. The following physical variables were also quantified in this study explosive distance (m; distance covered with an acceleration above 1.12 m/s^2^), number of very high-intensity accelerations (>+3 m/s^2^ with minimum duration of 0.5 s), number of very high-intensity decelerations distance (<−3 m/s^2^ with minimum duration of 0.5 s) [40].

### 2.4. Statistical Analysis

All data are presented as the mean ± standard deviation. The analysis was carried out with the software R, version 4.2.0 (R Foundation for Statistical Computing, Vienna, Austria). Linear mixed-effect models with random intercepts for individual players were used to assess the effects of team (first team/U-18), position, and their interaction on physical match performance data. When there was a significant effect of playing position, post-hoc Tukey comparisons were performed to check which positions were different. The differences between teams and positions were standardized by the between-subject standard deviation of each outcome variable to determine the effect size (ES). Standardized differences were evaluated as trivial (<0.2), small (0.2–0.6), moderate (0.6–1.2), large (1.2–2.0), and very large (2.0–4.0) [41]. The statistical significance was set at *p* < 0.05.

## 3. Results

**Figure 1:**
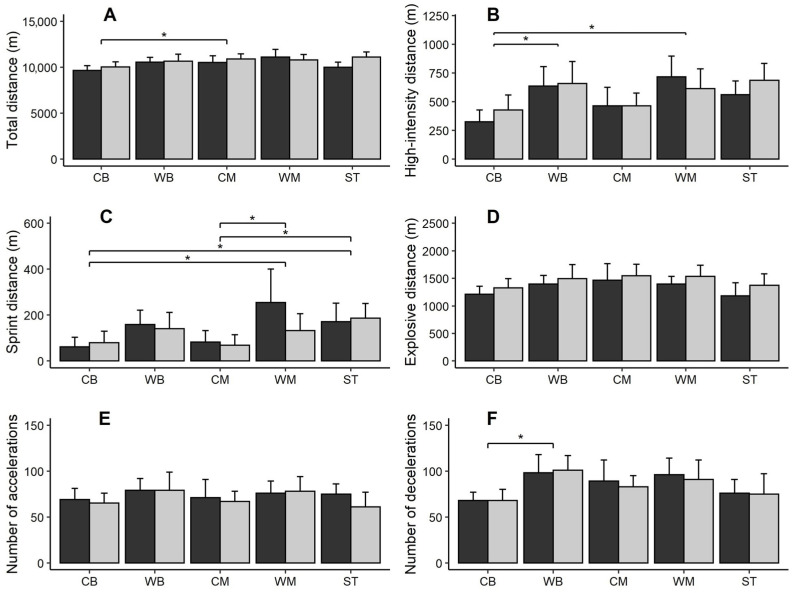
Mean of total distance (**A**), high-intensity distance (**B**), sprint distance (**C**), ex-plosive distance (**D**), number of accelerations (**E**), and number of decelerations (**F**) in first team and U-18 players. CB: Center backs; WB: Wing backs; CM: Central midfielders; WM: Wide midfielders; ST: Strikers. * denotes a significant difference between positions (*p* < 0.05).

The mean ± standard deviation values of all examined physical performance variables in first team and U-18 players divided by playing position are shown in Figure 1 for descriptive purposes.

Table 2 displays the differences between U-18 and first team players for the examined match physical performance variables as estimated by the linear mixed-effect regression analyses.

When compared to first team players, U-18 players showed trivially to moderately higher mean values for total distance covered, high-intensity distance, explosive distance, and number of decelerations, and lower mean values for sprint distance and number of accelerations, with small differences. The estimated differences between U-18 and first team players were not statistically significant (all *p* > 0.05) (Table 2). The linear mixed-effect regression analysis also revealed, for all the examined dependent variables, no significant effect (*p* > 0.05) of team by position interaction. There was also no significant effect of position for explosive distance and the number of accelerations (both *p* > 0.05). Conversely, there was a significant effect of playing position for total distance, high-intensity distance, sprint distance, and number of decelerations. On average, total distance was lower in CB compared to WM, with an estimated difference of 1297 m (ES = 2.30, very large; *p* = 0.01), while high-intensity distance was lower in CB compared to both WM (350 m, ES = 2.19, very large; *p* = 0.01) and WB (295 m, ES = 1.84, large; *p* < 0.01). Moreover, sprint distance was lower in CB compared to both ST (118 m, ES = 1.71, large; *p* = 0.03) and WM (167 m, ES = 2.42, very large; *p* = 0.01), and was lower in CM compared to both ST (104 m, ES = 1.51, large; *p* = 0.03) and WM (154 m, ES = 2.23, very large; *p* = 0.01). Finally, the number of decelerations was 36 accelerations (ES = 1.99, large; *p* = 0.03) lower in CB compared to WB.

## 4. Discussion

The aim of this study was to analyze a selection of physical match performance indicators of elite senior and youth soccer players from an SP club and compare the physical match performance between competition levels (senior versus youth), and identify any differences between playing positions. There were no actual differences found between first team and U-18 players for the examined physical performance variables, while some differences were reported between playing positions.

The lack of differences between both teams is the main novel finding of the present study, and to the best of the author’s knowledge, only one other study with a similar approach was found [24]. Specifically, that study compared U-17, U-19, and the first Danish teams. The authors showed a higher number of accelerations and decelerations for the U-19 team when compared with both the first and U-17 teams, while no differences were found for distance covered during high-intensity running or sprinting [24]. However, the present study only corroborated the non-differences between first and U-18 teams for high-intensity running and sprinting since the number of accelerations and decelerations did not differ between the first and U-18 teams. These findings may be justified with similar training characteristics for both teams and/or the specific characteristics of the SP, which should continue to be analyzed in future research to confirm the present results.

In addition, the U-18 players in the present study covered higher total distance than U-18 international players [42], U-18 England players [43], U-19 Croatian players [44], U-19 Italian players [45], U-20 Brazilian players [46], U-20 Italian players [47], and very similar total distance to U-18 England players [48]. Indeed, only two studies showed a higher total distance covered than the present study [49,50]. The present study also showed higher values for high-intensity and sprint distance compared with U-18 England players [43], U-19 Croatian players [44], and U-20 Italian players [47].

On the one hand, previous information highlighted that U-18 players from the present study showed higher levels of intensity. Although, the first team showed lower levels of total distance and high-intensity speed thresholds in matches than other studies conducted in first teams during training sessions, as highlighted by a recent systematic review [51]. This could be a concern considering that some studies showed that weekly training sessions did not express the same intensity as matches [52,53]. However, there is scant literature on SP teams to confirm if the present data can be generalized across all SP teams, thus, this would suggest more research is warranted. Nonetheless, the training methodology aimed at developing top physical performances at ~18 years, or the playing formation/style/strategy similarity between both first and U-18 teams from this club, may justify the current findings.

Regarding positional differences, the results for both teams showed an overall tendency of lower values for CB for total distance, sprint distance, and number of decelerations. In addition, WM showed a tendency for higher values in total distance, high-intensity distance, and sprint distance, while WB showed a tendency for higher values in accelerations and decelerations. Considering total distance, the present results are in contrast with previous studies that showed higher values for CM for adult professional, semi-professional, and amateur teams [2,16,54,55,56,57,58,59] and young soccer teams [60,61]. Such results can be justified by the nature and role of the position within the team, as well as the coaching strategy and/or game plan [62]. In fact, higher values were found for WM, although there was only one difference between WM and CB which seems to corroborate previous results found across 30 matches of the Spanish league and champions league [21]. Other studies in professional soccer players [2,59,60] showed that ST performed higher sprint distances, which is supported by the findings of the current study. This was also confirmed in studies examining young soccer players [47,62,63]. Beyond sprint distance, WM also showed a tendency for higher values in high-intensity distance, which was also supported by Ingebrigtsen et al. [27], that registered higher values for professional players in wide playing positions. This finding was also observed in young WM players which supports the present results [62,63].

Indeed, this study [2,59,60] also supported the higher values observed for WB when compared with CB regarding decelerations (the only difference reported). However, such findings opposed those previously stated by Modric et al. [16], who reported that CM performed more accelerations and decelerations, which was not the case in the present study. Furthermore, CB showed a tendency to produce lower values than other positions. Such a finding is in line with a previous study conducted by Modric et al. [56] that attributed such results to their technical role within the team (e.g., aerial duels, tackles, positioning, and interception of balls passed to the attackers). Finally, WB and WM are the playing positions that highlighted similarities. For instance, it was found that CM had the lowest number of decelerations [39], which is in line with our results.

## 5. Limitations

Only two teams from the same SP club were examined, which limited the total sample size and that of each position and thus may affect the generalization of results related to positions. Moreover, and despite not being an aim of the present study, internal load variables (e.g., rating perceived exertion) may provide more insights into the findings of the present study if applied. This variable was not included in the present study as it is not well documented in U-18 players. Therefore, it was not included to ensure it did not affect its validity.

The fact that U-18 players were compared with the first team may also be a limitation, previous studies have included other age brackets, such as U-19 or U-20 [26], which is suggested to consider in future studies. Such limitations can make it difficult to generalize the current results to male players of different age categories in different countries. Thus, a further suggestion for future research is to consider the previous limitations to analyze the other highlighted aspects. Finally, it is also suggested to consider the same type of comparison (e.g., first team versus U-18 team) for training data which could provide other details about training periodization.

## 6. Practical Applications

The highlights of this study support the analysis of positional match demands, which may provide useful information for training program design and tactical strategy. For example, the differences observed between playing positions in match-play suggest that the position specificity approach should be taken in consideration for training periodization. Furthermore, this study is relevant by showing similar external load values of two teams from the same club at different levels. This may also be used as a strategy when deciding when to include young soccer players into the first team.

## 7. Conclusions

This study confirmed that playing position influenced the external load performance in soccer match-play. However, it failed to support the hypothesis that suggested the competitive level could also influence the external load, which, in this case, was not evident when comparing first team and U-18 players.

## Figures and Tables

**Table 1:** Age, anthropometric, and number of players per playing position in the two examined squads.

				Number of Players				
Squad	Age (mean ± SD)	Body-mass (mean ± SD)	Height (mean ± SD)	CB *n* = 8	WB *n* = 8	CM *n* = 9	WM *n* = 6	ST *n* = 7
First team (*n* = 22)	25.9 ± 4.5 years	78.3 ± 8.2	1.82 ± 0.07 m	*n* = 5	*n* = 5	*n* = 6	*n* = 2	*n* = 4
U-18 youth (*n* = 16)	16.8 ± 0.9 years	70.1 ± 6.8 kg	1.77 ± 0.06 m	*n* = 3	*n* = 3	*n* = 3	*n* = 4	*n* = 3

**Table 2:** Estimated differences for the examined physical performance variables between first team and U-18 players.

Dependent Variable	Estimated Difference (U-18 Minus First Team) with 95% CI	*p*-Value	ES
Total distance (m)	223 (−653 to 1100)	0.61	0.40 (small)
High-intensity distance (m)	61 (−171 to 292)	0.60	0.38 (small)
Sprint distance (m)	−4 (−107 to 100)	0.94	0.06 (trivial)
Explosive distance (m)	178 (−210 to 567)	0.35	0.78 (moderate)
Number of accelerations	−5 (−31 to 21)	0.70	0.38 (small)
Number of decelerations	6 (−27 to 40)	0.71	0.33 (small)

CI: Confidence interval; ES: Effect size.

## Data Availability

Data will be provided to all interested parties upon reasonable request.
